# Quantitative comparison of EST libraries requires compensation for systematic biases in cDNA generation

**DOI:** 10.1186/1471-2105-7-77

**Published:** 2006-02-17

**Authors:** Donglin Liu, Joel H Graber

**Affiliations:** 1 The Jackson Laboratory, 600 Main Street, Bar Harbor, ME 04609, USA

## Abstract

**Background:**

Publicly accessible EST libraries contain valuable information that can be utilized for studies of tissue-specific gene expression and processing of individual genes. This information is, however, confounded by multiple systematic effects arising from the procedures used to generate these libraries.

**Results:**

We used alignment of ESTs against a reference set of transcripts to estimate the size distributions of the cDNA inserts and sampled mRNA transcripts in individual EST libraries and show how these measurements can be used to inform quantitative comparisons of libraries. While significant attention has been paid to the effects of normalization and substraction, we also find significant biases in transcript sampling introduced by the combined procedures of reverse transcription and selection of cDNA clones for sequencing. Using examples drawn from studies of mRNA 3'-processing (cleavage and polyadenylation), we demonstrate effects of the transcript sampling bias, and provide a method for identifying libraries that can be safely compared without bias. All data sets, supplemental data, and software are available at our supplemental web site [1].

**Conclusion:**

The biases we characterize in the transcript sampling of EST libraries represent a significant and heretofore under-appreciated source of false positive candidates for tissue-, cell type-, or developmental stage-specific activity or processing of genes. Uncorrected, quantitative comparison of dissimilar EST libraries will likely result in the identification of statistically significant, but biologically meaningless changes.

## Background

Expressed sequence tags(ESTs) are single strand reads of transcribed sequence generated from cDNA clones [2-6]. EST sequencing typically originates in the vector, and can include either 5'- or 3'-terminal sequence of the cDNA clone. ESTs have historically provided data for gene discovery [7-11], tissue- or stage-specific gene expression [12-15], alternative splicing [16,17], and alternative polyadenylation [4,5,18-21].

While EST-based gene discovery can be quite successful, the wide dynamic range of mRNA abundance and the cost of EST creation led to the development of procedures such as normalization and subtraction [13,22], which increase the likelihood of sampling rare or tissue-specific transcripts, at the cost of lost quantitative relationships between different transcripts in a library. Normalization and subtraction utilize a common mechanism, which can be briefly described as heat dehybridization of cDNA, rapid re-hybridization in the presence of a 'driver' sample, extraction of the double-stranded portion of the sample, and finally sequencing of a sampling of the remaining single-stranded sequences. The rapid re-hybridization step favors duplex formation of abundant species, therefore the remaining single-stranded sample is enriched for rare transcripts. The difference between normalization and subtraction lies in the choice of driver sequences. In normalization, the drivers come from the same sample, whereas in subtraction, the driver comes from a separate sample (or even pool of samples).

Since cDNA clones are created from mRNA sequences, the distribution of ESTs in a non-normalized library is presumably reflective of the population of mRNA sequences in the originating tissue. Audic and Claverie [12] demonstrated how non-normalized EST libraries could be analyzed to generate "transcript profiles" or "digital Northerns," and further developed rigorous statistical tests for significant variation between tissue or cell types. Several methods have been subsequently developed to enable studies of cDNA libraries to elucidate targets and mechanisms of tissue- and/or stage-specific gene expression [23-25]. Bioinformatic tools, such as Tissuelnfo [26], BodyMap [27] and ExQuest [28] utilize counts of ESTs in libraries for high-throughput identification of tissue expression profiles and specificity, in spite of the known limitations on their fidelity of representation of gene expression on the originating tissue [7].

cDNA library generation is dependent on several steps, including selection and preparation of tissue, RNA purification, RNA to cDNA conversion, and cloning and transformation of the cDNA [29]. Procedures such as reverse transcription and selection of cDNA clones for sequencing can introduce systematic biases in any quantitative analysis using cDNA libraries. Confounding matters further, the depth of annotation among EST libraries is not uniform and often incomplete. It is worth noting that differences in transcript sampling between disparate laboratories and research groups are not unexpected, given the varied motivations and resources of the library creators.

We present here a method and means to identify and compensate for the biases in transcript sampling and enable quantitative comparisons between libraries. We analyzed and quantized over 900 mouse EST libraries with at least 100 entries, estimating the length distributions of cDNA inserts and their originating mRNA transcripts through alignment to a reference set of transcripts. (Similar analyses are being prepared for other organisms and will be made available on our web site [1].) The cDNA insert length distribution provides information about the efficiency and/or goals of the reverse transcription reaction, while the transcript length distribution evaluates the variety of transcripts sampled in the library. Our results show that the combined steps of reverse transcription and selection of clones (including optional restriction by insert size) for sequencing can introduce significant biases into transcript sampling in EST libraries, but that through identification and characterization of these biases, quantitative comparisons can be enabled. We identified the systematic biases as a part of a study of alternative 3'-processing in mouse spermatogenesis [Liu et al., in preparation], and therefore present our analysis with examples related to 3'-processing. The biases we describe are equally applicable, however, to any quantitative comparative analysis between distinct EST libraries, including assessment of tissue specificity of gene expression or processing.

## Results and discussion

**Figure 1:**
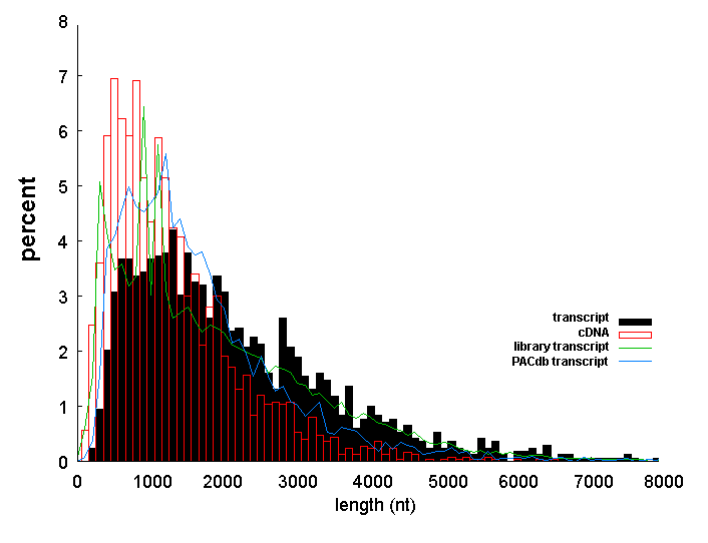
**Example estimates of cDNA insert and mRNA transcript length distributions for an EST library**. The estimated length distributions of cDNA inserts (red bars) and sampled transcripts (black bars) in a mouse EST library generated from round spermatids (McCarrey, J., Eddy, M. et al, unpublished data). For comparison, the length distributions of the ENSEMBL [44] and PACdb [21] reference transcripts are plotted in blue and green, respectively.

Comparative studies of EST libraries, such as those to estimate tissue- or stage-specificity of expression, typically avoid the use of normalized or subtractive libraries. We find, however, that even non-normalized libraries are subject to systematic biases (arising from the procedures used in their generation) that can distort quantitative studies. To quantify these biases, we aligned all ESTs from each library to a reference transcript set, and used the results to estimate the length distributions of the cDNA inserts and sampled transcripts. A typical result is shown in Figure 1. A significant fraction of the libraries examined show the roughly lognormal distribution of both cDNA insert and transcript lengths, as shown in Figure 1. Comparison of the cDNA insert and transcript length distributions for a given library can give insights into the goals or parameters of the library generation. Similar distributions are indicative of an attempt to generate full length transcripts. A tight (low-variance), short, distribution of cDNA insert lengths indicates a fixed, relatively short reverse transcription reaction that results in cDNA insert lengths essentially independent of the transcript length. (Interestingly, such libraries are typically relatively unbiased in transcript sampling when compared with the reference sets). A tight distribution of cDNA insert lengths longer than a few hundred nucleotides indicates a size selection step of either mRNA or cDNA sequences prior to sequencing.

**Figure 2:**
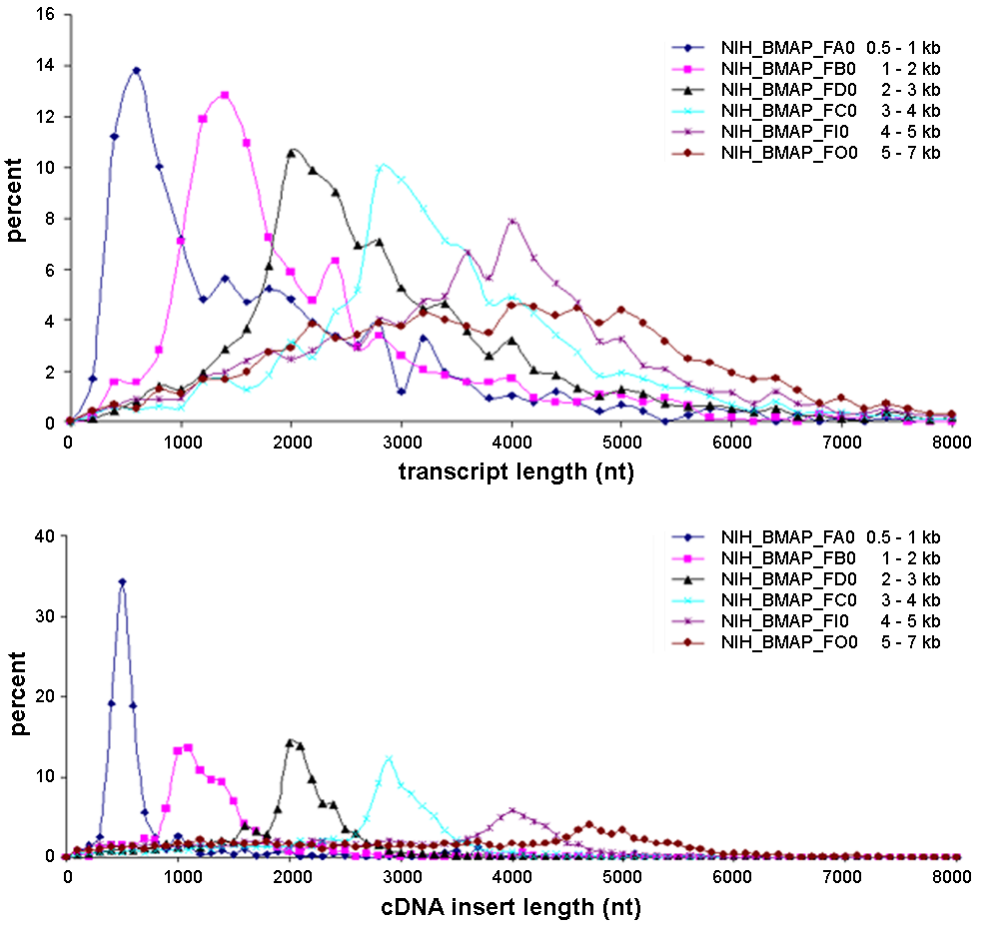
**Variation in cDNA insert and transcript length distributions for EST libraries from a common sample**. Estimated transcript (top panel) and cDNA insert (bottom panel) length distributions for several EST libraries derived from a common tissue sample [30]. These libraries were explicitly created to generate full-or near full-length cDNA sequences, and then size selected into the approximate ranges shown in the legends.

We examined several groups of libraries generated from a common tissue sample [30], described in Table 1. These specific libraries were generated in the NIH Brain Molecular Anatomy Project (BMAP) [30], the goal of which was generation of a large number of full-length transcripts from the mouse nervous system. Together these libraries represent a broad sampling of mRNA transcripts between 500 and 7000 nucleotides in length, with different libraries putatively selected to contain a specific size range. Figure 2 displays our estimates of the length distributions for the transcripts (top panel) and cDNA inserts (bottom panel) for these libraries. As expected, the modes of the cDNA insert and transcript length distributions correlate quite well with the targeted ranges of the EST libraries. The attempt at creation of full-length transcripts in these libraries is apparent in comparison of the two panels in Figure 2, and also in the fourth column of Table 1, which shows the L-Divergence measurement between cDNA insert and transcript distributions for each library.

**Table 1:** Analysis of a group of EST libraries from a common sample. Each of these EST libraries was generated from a common tissue sample. Distance is calculated as L-divergence (Equation 3) between distributions of cDNA and transcript lengths for each library. The targeted size range of the cDNA inserts for each library ranges from 0.5 k to 7 k as described previously [30]

LibName	Total ESTs	5' ESTs	L-Divergence	Targeted size(kb)
NIH-BMAP-FA0	2519	2352	0.308	0.5–1
NIH-BMAP-FB0	1092	994	0.199	1–2
NIH-BMAP-FD0	6594	5812	0.191	2–3
NIH-BMAP-FC0	5969	3458	0.154	3–4
NIH-BMAP-FI0	6769	5778	0.109	4–5
NIH-BMAP-FO0	6135	5109	0.077	5–7

Interestingly, for the libraries with the shorter targeted lengths (*e.g*., libraries NIH-BMAP-FAO and NIH-BMAP-FBO), our analysis indicates that the majority of the cDNA inserts and sampled transcripts come from the targeted range, however, as the targeted range increases, this specificity decreases, and a significant number of transcripts with length apparently outside the targeted range are found. This increasing discrepancy could arise from a number of effects. Electrophoretic separation is imprecise, including imperfections in the gels used for size selection and anomalous migration times for specific transcripts. Such unpredictable behavior will more significantly affect measurements of large molecules. Large molecules are also comparatively more prone to breakage. Our method for assignment of EST to transcripts is also a likely source of some ambiguity, as it is designed to give a reasonable picture of the characteristics of the complete library, but is certainly not guaranteed to give exact results for any specific EST or transcript.

**Figure 3:**
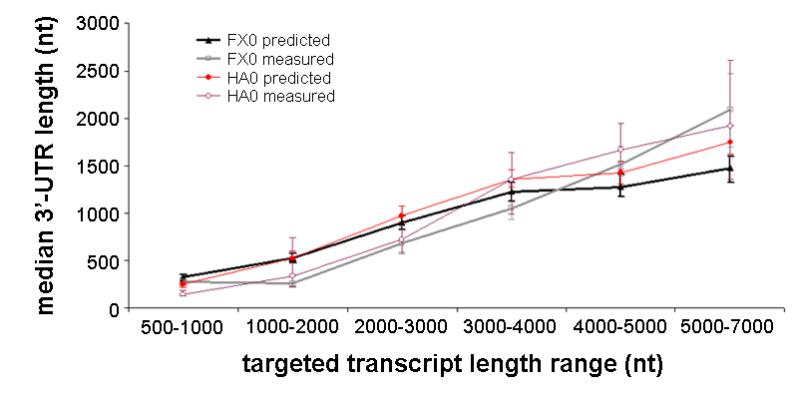
**Comparison of measured and expected median 3'-UTR lengths in EST libraries from a common sample**. A comparison of empirically derived and expected median 3'-UTR length for two groups of related EST libraries. Each set contains 6 libraries from a common tissue sample and targeted transcripts range from 0.5–1 k, 1–2 k, 2–3 k, 3–4 k, 4–5 k and 5–7 k. Expected 3'-UTR length distributions were calculated as described in Methods. The FX0 and HA0 library sets represent EST libraries from pools of tissues from brain and eye, respectively [30]. The correlations between the measured and predicted median 3'-UTR lengths for the FX0 and HA0 sets are 0.935 and 0.982, respectively.

mRNA 3'-processing (cleavage and polyadenylation) and the corresponding specification of 3'-UTR in eukaryotes play an important role for mRNA localization, translational efficiency, and stability [31-35]. We use EST-to-genome alignments to identify and characterize 3'-processing sites and 3'-UTRs [21]. Our studies of alternative 3'-processing during mouse spermatogenesis (Liu et al., in preparation) indicated that the distribution of 3'-UTR lengths can systematically change as a function of tissue type or growth conditions. Our analysis of PACdb [21] makes it clear that 3'-UTR length is dependent on transcript length (see supplemental materials), therefore accurate characterization of these changes requires that we identify and compensate for the variation that arises due to differences in transcript sampling, as described above. As described in Methods, we generate an expected distribution of 3'-UTR lengths for any given library based on random sampling of (1) the transcript length distribution of the library, and (2) the distribution of 3'-UTR lengths as a function of transcript length. In Figure 3, we compare the measured and expected median 3'-UTR lengths (including ninety-five percent confidence intervals) for two additional groups of EST libraries, generated as size selected ranges from a common tissue in the Brain Molecular Anatomy Project [30]. The FX0 set, generated in a pool of brain tissues, includes libraries NIH-BMAP-FV0, NIH-BMAP-FX0, NIH-BMAP-FR0, NIH-BMAP-FW0, NIH-BMAP-FY0 and NIH-BMAP-GI0, while the HA0 set, generated from eye tissues, consists of libraries NIH-BMAP-HC0, NIH-BMAP-HA0, NIH-BMAP-GZ0, NIH-BMAP-HD0, NIH-BMAP-HB0 and NIH-BMAP-HE0. The agreement is quite good, with measured-expected correlations calculated at 0.935 and 0.982 for the FX0 and HA0 sets, respectively. Interestingly, the difference between measured and expected 3'-UTR lengths is greatest in library NIH-BMAP-GI0, which represents the longest transcripts in the sample of pooled brain tissues. In preliminary studies, we have found (data not shown) that brain tissues may, in fact, be subject to a selection for longer 3'-UTR sequences.

From the analysis presented in Figure 3, we conclude that with care, quantitative comparisons between EST libraries can be used for the identification of tissue- or stage-specific phenomena, however, systematic variations in transcript sampling must be controlled. We explicitly identify EST libraries that can be safely compared without this bias by using the L-Divergence (Equation 3) to compare the estimated transcript length distributions of all pairs of libraries. Quantitative comparison can be made between pairs of EST libraries whose divergence is less than an empirically determined threshold value. To facilitate such analyses by other investigators, we have made available our tools, data, and results on a web server [1]. Included in this package is a tool that will take as input a single EST library identifier and return a list of other libraries for which quantitative comparisons can safely be made.

While our examples have come from studies of mRNA 3'-processing, the systematic issues we have identified can bias similar analyses of phenomena such as tissue-specific changes in either global transcription patterns of many genes or processing of a specific gene. As long as the measured quantity has a dependence on (or causes a variation in) the transcript size, the effects we describe here can result in a false positive identification of a statistically significant, but biologically meaningless change. As a simple example, consider a gene with a 5000-nucleotide long transcript. If the relative expression level for this gene was compared between two EST libraries whose transcript size sampling resembled libraries NIH-BMAP-FA0 and NIH-BMAP-FO0, respectively, we would expect a large discrepancy favoring the latter library, even if the true expression level in the two tissues was identical.

**Figure 4:**
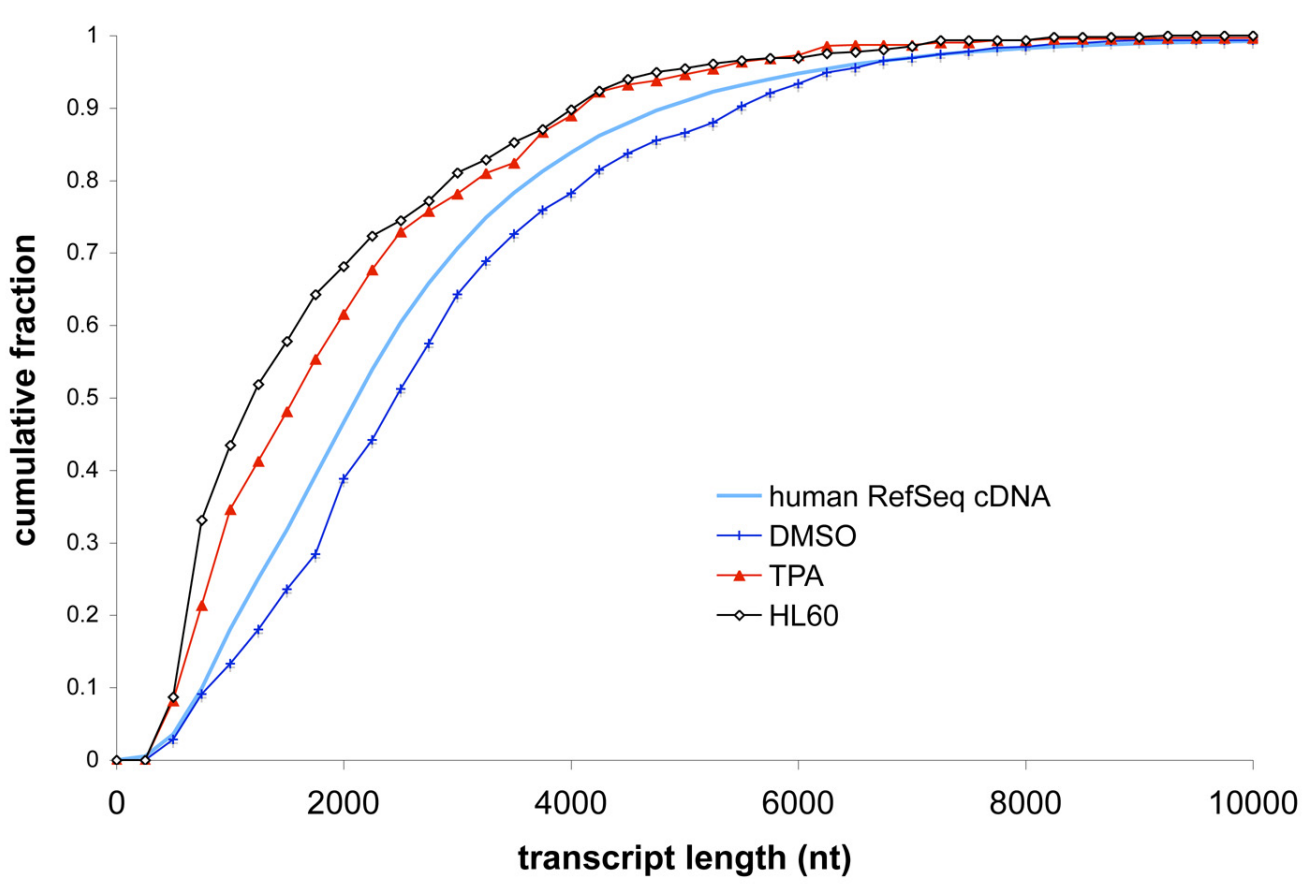
**Estimated cumulative length distributions of the transcripts sampled by the HL60, DMSO-induced granulocytoid, and TPA-induced monocytoid cell lines [36]**. For comparison, the cumulative length distribution of the human RefSeq cDNA collection [37] is also shown.

To further investigate the effect on measurement of gene expression, we examined the EST libraries originally generated by Okubo *et al*. [36] and later used as examples by Audic and Claverie [12]. These libraries consist only of 3'-ESTs, so we can assess the sampled transcript length distribution but not the insert length distribution. Briefly, these include three libraries, one generated from an HL60 human promyelocytic cell line, and two derived cell lines, a DMSO-induced granulocytoid cell line and a TPA-induced monocytoid cell line. In both the original and subsequent analyses, a number of genes were identified as differentially expressed between the cell types. Using the human RefSeq cDNA collection [37] as a reference, we generated transcript length distributions (Figure 4). Intriguingly, the DMSO-induced granulocytoid library appears to have sampled transcripts with a significantly longer length distribution than either of the other two libraries, which, in contrast, follow distributions quite similar to each other. Our analysis indicates the possibility that a portion of the genes identified as differentially expressed are false positives. Okubo *et al*. experimentally verified a number of genes as differentially expressed [36], however their measurements were on double-stranded cDNA saved at an intermediate stage of library construction. This brings up two distinct possibilities: either the list of differentially expressed genes includes a non-zero number of false positives due to differences in sampling of the transcripts during library construction, or the change in expression pattern upon DMSO-induced differentiation includes a systematic shift to significantly longer transcripts. Our analysis does not question the computational approaches of either the original [36] or subsequent manuscript [12], but rather the required assumption that the two originating samples are *identical *in preparation.

It is clear from our analysis that the common practice of computationally pooling EST libraries from a common tissue type or developmental stage, while increasing the gene coverage, is not likely to accurately reproduce relative expression levels of the original tissue sample. To see this, consider that our results indicate that in nearly all unbiased samples, the underlying transcript length distribution follows a roughly lognormal distribution, similar to the PACdb transcript, in Figure 1. As we have shown, distinct EST libraries will sample from this underlying distribution in a manner determined by preparation of the library. Simply combining disparate samples together will almost certainly distort the relative contributions of the different size transcripts. For example, the three groups of Brain Map libraries shown in Table 1 and Figure 3 represent roughly equal size samples from six different size ranges of transcripts, which if pooled, would oversample long (>3000 nucleotides) transcripts at the expense of shorter ones.

## Conclusion

EST libraries contain a wealth of data regarding gene expression and specifics of transcript processing across a broad range of tissue- and cell-types and developmental stages. These data have been collected, however, by a wide range of researchers with varied procedures and goals, making large-scale comparative studies using these sequences problematic. The level of detail in the annotation regarding preparation techniques is not uniform, and in many cases incomplete (especially with regard to whether or not clones were size-selected). Even in libraries prepared without the two best understood systematics, normalization and subtraction, we find systematic variations in the sampling of transcripts. We have shown that estimates of the cDNA insert and originating transcript length distributions can be used to assess and compensate for systematics of library generation and enable quantitative analysis. Our tools and analyses are available via a web server [1] to help other researchers separate truly biologically meaningful changes in gene expression or processing from those that arise due to systematic biases.

## Methods

We downloaded the NCBI dbEST (release 030405, 03/04/2005) [38], and extracted 935 mouse libraries from a variety of tissues and organs, for a total of approximately 4.3 million ESTs. We used several sources for transcript reference sets, including 26,000 sequences from NCBI's mouse RefSeq transcript set [37], 20,515 non-redundant transcript sequences from ENSEMBL, version 27.33c [39], and the set of 39,000 transcripts implied by all putative mouse 3'-processing sites in PACdb [21]. Since EST libraries can contain contaminant sequences [40], we filtered and eliminated ESTs with evidence of vector/linker, *E.coli*, mitochondrial, or non-protein-coding RNAs. The filtered EST sequences were subsequently aligned to the reference transcripts using BLAT [41]. BLAT was chosen based on execution and ease of use, however, since we are looking for very high quality alignments of ESTs to mRNA sequences, the alignment problem is conceptually quite easy, and the choice of alignment tool is essentially immaterial. Each EST-transcript alignment was ranked in terms of quality and EST coverage, as defined in Equation 1 and 2.

*alignmentScore *= *m *- *n *- *g *    (1)

coverageOnEST=alignedLengthOnESTESTLength     (2)
 MathType@MTEF@5@5@+=feaafiart1ev1aaatCvAUfKttLearuWrP9MDH5MBPbIqV92AaeXatLxBI9gBaebbnrfifHhDYfgasaacH8akY=wiFfYdH8Gipec8Eeeu0xXdbba9frFj0=OqFfea0dXdd9vqai=hGuQ8kuc9pgc9s8qqaq=dirpe0xb9q8qiLsFr0=vr0=vr0dc8meaabaqaciaacaGaaeqabaqabeGadaaakeaaieGacqWFJbWycqWFVbWBcqWF2bGDcqWGLbqzcqWGYbGCcqWGHbqycqWGNbWzcqWGLbqzcqWGpbWtcqWGUbGBcqWGfbqrcqWGtbWucqWGubavcqGH9aqpdaWcaaqaaiabdggaHjabdYgaSjabdMgaPjabdEgaNjabd6gaUjabdwgaLjabdsgaKjabdYeamjabdwgaLjabd6gaUjabdEgaNjabdsha0jabdIgaOjabd+eapjabd6gaUjabdweafjabdofatjabdsfaubqaaiabdweafjabdofatjabdsfaujabdYeamjabdwgaLjabd6gaUjabdEgaNjabdsha0jabdIgaObaacaWLjaGaaCzcamaabmaabaGaeGOmaidacaGLOaGaayzkaaaaaa@652C@

where *m*, *n*, and *g *denote the count of matched, mismatched, and gapped positions, respectively, in the alignment. We retained only alignments for which *coverageOnEST *was greater than or equal to 0.9. If more than one reference transcript was aligned above this threshold, the alignment with the best *alignmentScore *was selected. In case of a tie, one transcript was selected at random.

**Figure 5:**
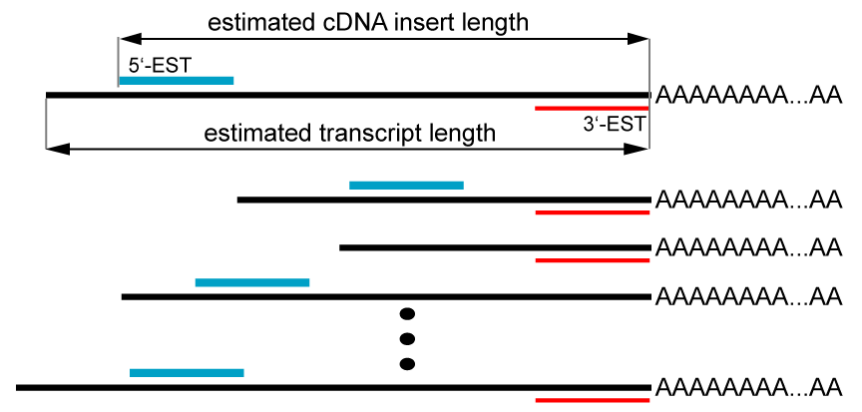
**Estimation of transcript and cDNA insert lengths through EST to reference transcript alignments**. Using EST-to-transcript alignments to estimate cDNA insert and originating transcript length distributions for EST libraries. 5'-EST alignment positions are used to determine the size of cDNA inserts. Reference transcripts matched by either 5'- or 3'- ESTs are used to estimate the sampled transcript lengths. Black lines represent polyadenylated mRNA transcripts, and 5'-ESTs and 3'-ESTs are represented in blue and red, respectively.

To estimate the cDNA insert and originating transcript length distributions, we collected information as shown in Figure 5. We identified 5'- and 3'-ESTs as those that aligned in sense and anti-sense, respectively with the reference transcript, with care to identify libraries in which the reported sense of the EST was conceptually reverse-transcribed. The distance from 5'-EST starting points to the 3'-end of the matched transcripts was used to estimate the cDNA insert size, while the transcript length distribution was estimated by collecting the lengths of all reference transcripts matched by either 5'- or 3'-ESTs. An example of transcript and cDNA insert length distributions is presented in Figure 1. This analysis explicitly ignores randomly primed libraries. However, such libraries represent a relatively small fraction of the total (less than ten mouse libraries in dbEST are explicitly labeled as randomly primed). In addition, even if a library was randomly primed, it would affect our estimate of the cDNA insert size, but not the transcript size, on which our comparative analysis is based.

For comparison of distributions, many metrics are available, including Euclidean distance, and Pearson or Spearman Correlation. We used a normalized L-Divergence [42], which is based on Shannon's entropy and is defined in Equation 3:

L=12∑ip1(i)logp1(i)p1(i)+p2(i)2+p2(i)logp2(i)p1(i)+p2(i)2     (3)
 MathType@MTEF@5@5@+=feaafiart1ev1aaatCvAUfKttLearuWrP9MDH5MBPbIqV92AaeXatLxBI9gBaebbnrfifHhDYfgasaacH8akY=wiFfYdH8Gipec8Eeeu0xXdbba9frFj0=OqFfea0dXdd9vqai=hGuQ8kuc9pgc9s8qqaq=dirpe0xb9q8qiLsFr0=vr0=vr0dc8meaabaqaciaacaGaaeqabaqabeGadaaakeaacqWGmbatcqGH9aqpdaWcaaqaaiabigdaXaqaaiabikdaYaaadaaeqbqaaiabdchaWnaaBaaaleaacqaIXaqmaeqaaOGaeiikaGIaemyAaKMaeiykaKcaleaacqWGPbqAaeqaniabggHiLdacbiGccqWFSbaBcqWFVbWBcqWFNbWzdaWcaaqaaiabdchaWnaaBaaaleaacqaIXaqmaeqaaOGaeiikaGIaemyAaKMaeiykaKcabaWaaSaaaeaacqWGWbaCdaWgaaWcbaGaeGymaedabeaakiabcIcaOiabdMgaPjabcMcaPiabgUcaRiabdchaWnaaBaaaleaacqaIYaGmaeqaaOGaeiikaGIaemyAaKMaeiykaKcabaGaeGOmaidaaaaacqGHRaWkcqWGWbaCdaWgaaWcbaGaeGOmaidabeaakiabcIcaOiabdMgaPjabcMcaPiab=XgaSjab=9gaVjab=DgaNnaalaaabaGaemiCaa3aaSbaaSqaaiabikdaYaqabaGccqGGOaakcqWGPbqAcqGGPaqkaeaadaWcaaqaaiabdchaWnaaBaaaleaacqaIXaqmaeqaaOGaeiikaGIaemyAaKMaeiykaKIaey4kaSIaemiCaa3aaSbaaSqaaiabikdaYaqabaGccqGGOaakcqWGPbqAcqGGPaqkaeaacqaIYaGmaaaaaiaaxMaacaWLjaWaaeWaaeaacqaIZaWmaiaawIcacaGLPaaaaaa@71F1@

where *p*_1 _and *p*_2 _denote the two density distributions being compared. This metric is bounded between 0 and 1, and furthermore has shown to be robust under a wide variability in probability distributions [42,43]. To characterize library-specific changes in 3'-processing, we used the ESTs from each library to identify probable 3'-processing sites as described previously [21]. Projected 3'-UTR lengths were calculated by measuring the genomic separation between the 3'-processing site and the stop codon of the assigned gene. Putative 3'-processing sites for a given EST library are computationally normalized, such that all statistical analysis (*e.g*., 3'-UTR length distributions) is performed on the set of unique sites, with no weight given to EST copy numbers.

We used an empirically determined distribution of 3'-UTR lengths as a function of transcript length (available in the supplement) as part of a two-step sampling process to generate an expected 3'-UTR length distribution for each mouse EST library. We first randomly sampled transcript lengths according to the library's estimated distribution, which was determined as shown in Figure 5. For each transcript length drawn, a second random draw was made from the 3'-UTR length distribution indicated from the PACdb data [21].
